# Helpful if “Medication took up more space in the course!” – a mixed methods study about pharmacotherapeutic knowledge and digital quizzes for learning and assessment in the medical programme

**DOI:** 10.1186/s12909-026-09292-7

**Published:** 2026-04-30

**Authors:** SM Wallerstedt, K Jood, S Steingrimsson, E Wentz, J Zelano, F Bergquist

**Affiliations:** 1 Department of Pharmacology, Sahlgrenska Academy, University of Gothenburg, Gothenburg, Sweden; 2 Center for Health Technology Assessment, Sahlgrenska University Hospital, Region Västra Götaland, Gothenburg, Sweden; 3 Department of Clinical Neuroscience, Sahlgrenska Academy, University of Gothenburg, Gothenburg, Sweden; 4 Department of Neurology, Sahlgrenska University Hospital, Gothenburg, Sweden; 5 Department of Psychiatry and Neurochemistry, Sahlgrenska Academy, University of Gothenburg, Gothenburg, Sweden; 6 Department of Psychiatry, Sahlgrenska University Hospital, Gothenburg, Sweden

**Keywords:** Medical programme, Neurology, Pharmacology, Psychiatry, Students

## Abstract

**Background:**

Pharmacotherapy and prescribing are core skills for physicians, and all medical graduates must master the basics. From a medical education perspective, it is important to understand factors that help students attain sufficient skills for safe and effective prescribing. In this study, we evaluated autograded pharmacotherapeutic quizzes for practice and summative assessments in two undergraduate clinical courses and explored medical students’ views on educational components they considered helpful in learning to treat patients with medications.

**Methods:**

Pharmacotherapeutic quizzes were implemented in two steps across two course instances for two clinical courses (Psychiatry and Neurology, seventh semester in the medical programme at the University of Gothenburg, Gothenburg, Sweden). In step I, voluntary practice quizzes and a summative assessment test were introduced. In step II, clinical contexts for the quiz questions were provided, and the summative test was expanded. The students’ achieved level of knowledge post-course was investigated before and after each step, using an anonymous voluntary knowledge evaluation test including 20 case-based single best answer (SBA) questions. Based on free-text replies to a concurrent questionnaire on students’ views on learning pharmacotherapy, a manifest content analysis was performed, guided by the research question “What in their education do medical students consider important in enabling them to treat patients with medications?” Meaning units were extracted, and emergent categories and themes identified.

**Results:**

In total, 274 out of 404 course participants took the knowledge evaluation test and completed the questionnaire (response rate: 68%; 56% women; 66% ≤24 years old). Compared with pre-quiz results (median correct answers out of 20 SBA questions = 10 (lower to upper quartile 9–13)), no difference was seen after step I (11 (8–13) correct answers; *P* = 0.88) but a clear improvement was seen after step II (14 (12–16); *P* < 0.0001). In the qualitative analysis, four themes emerged: *Curriculum*, *Clinical placement*, *Theoretical teaching* and *Student responsibility*. The second theme, including the categories *Preparation*, *Participation* and *Performance*, was particularly prominent.

**Conclusions:**

Elaborated quizzes about medications, for practice and summative assessment, may increase pharmacotherapeutic knowledge in medical students. The four emergent themes regarding what students consider important can guide future course developments.

**Supplementary Information:**

The online version contains supplementary material available at 10.1186/s12909-026-09292-7.

## Background

Pharmacotherapy and prescribing are core skills for physicians, and all students graduating from the medical programme must demonstrate competence in these areas. However, studies have reported that there is room for improvements regarding medical students’ knowledge in these fields [1, 2]. Furthermore, most final-year medical students report that they are not confident in their knowledge about basic pharmacology, including, for instance, pharmacodynamics and pharmacokinetics, of commonly used medications [2]. From a medical education standpoint, it is important to identify interventions that effectively facilitate medical students’ learning in pharmacotherapy, and to understand which educational components are useful for students to attain sufficient skills for safe and effective prescribing.

Educational activities in teaching prescribing have been reported to typically involve didactic lectures and group exercises [3]. However, this approach requires substantial and continuous efforts by teachers. For this reason, e-learning, another common educational intervention in the field [4], may be appealing. Among other e-learning interventions for improved knowledge, students have suggested quizzes [5]. Digital quizzes hold some practical advantages as an educational intervention. First, they require minimal teaching resources once implemented, in particular if the quizzes are autograded and the source of information is rarely updated, such as, in the case of pharmacology, the summary of product characteristics (SPC). Second, they can be used at any time by the students, for instance while waiting between patient-related activities during clinical placements, and between other educational activities.

Quiz-based learning enhances retention by engaging retrieval processes and providing corrective feedback [6]. Therefore, we decided to implement pharmacotherapy quizzes on the digital learning platform for both practice and summative assessment in two undergraduate clinical courses in medical education, while simultaneously evaluating the extent to which this approach could improve students’ knowledge levels. Concurrently, we explored medical students’ views on components within the medical programme that they considered helpful for acquiring the skills needed to treat patients with medications.

## Methods

This mixed methods study involved the development of autograded quizzes focused on medications relevant to the undergraduate clinical Psychiatry and Neurology courses in the medical programme. The quizzes were designed for both practice and summative assessment on the digital learning platform. Before implementation and after each development step, students who had just finished a course were invited to complete an anonymous knowledge evaluation test and a questionnaire. These tests and questionnaires formed the basis for quantitative and qualitative analyses. Some of the quantitative results have been briefly reported in Swedish [7].

### Setting

The study was conducted within the 5.5-year medical programme at the University of Gothenburg, Gothenburg, Sweden. The programme consists of 2 years focused mainly on preclinical medical science, followed by 3.5 years of clinical education including direct patient care. After the medical degree, a 1.5-year foundation internship with a limited prescribing licence must be completed before a full medical licence is obtained. At the time of this study, basic pharmacology was taught during the second semester and integrated in the Physiology course. The basic pharmacology part spanned 5 weeks corresponding to 7.5 European Credit Transfer System (ECTS) credits. Clinical pharmacology was taught for about 1 week primarily in the sixth semester, and pharmacotherapy and prescribing skills were expected to be acquired during the remainder of the medical programme. Most of the practical prescription training took place during the supervised 1.5-year internship. All Swedish universities, including the University of Gothenburg, are currently in the process of implementing a 6-year medical programme where students obtain full prescribing rights upon graduation, without a prior internship where prescribing is practised.

The study took place during the Psychiatry and Neurology courses in the seventh semester. These courses ran in parallel, with half of the students attending one course and the other half attending the other, before switching. The students taking part in this study participated in their first course of the semester, i.e., either Psychiatry or Neurology. Each course lasted 7 weeks (10.5 ECTS credits) and included a combination of traditional lectures, case-based seminars and clinical placements. In the Psychiatry course, the clinical placements were primarily in inpatient wards, whereas in the Neurology course, the clinical placements included both in- and outpatient settings.

### Quizzes for practice and summative assessment

The aim of the autograded digital quizzes was to reinforce theoretical pharmacology knowledge and encourage its application in clinical pharmacotherapy contexts. The quizzes focused on medications identified as particularly relevant for each course and listed at the beginning of the course. The questions―sometimes framed in patient cases―concerned mechanisms of action; pharmacokinetics, including dosing and considerations related to kidney function; clinical indications and treatment choices within each therapeutic area; adverse effects and drug–drug interactions; and medications in pregnancy. To reflect clinical practice, the quizzes were open-book. The quizzes were implemented in two steps, reflecting an iterative improvement approach; details are provided in Additional file 1, Table S1. The practice quizzes and the summative assessment test did not contain the same questions.

In step I, voluntary digital practice quizzes and a mandatory digital summative assessment test were introduced. The practice quizzes were available throughout the course, and the summative test was available during the final 2 weeks of the course and consisted of ten randomly selected true/false statements per attempt (unlimited number of attempts allowed). The statements were drawn from pools of 265 (psychiatry) or 157 (neurology) statements covering seven and five subject areas, respectively. Eight correct answers were required to pass the test.

Step II was added after evaluating the preliminary results from step I, which did not meet expectations for improved performance on the knowledge evaluation test (see the section “Evaluation” below). In step II, the digital quizzes were redesigned using the structure of the WHO 1994 Guide to Good Prescribing compiled by De Vries et al. [8] and clinical contexts were added. Furthermore, enhanced efforts were put into constructive alignment, i.e., with increased linkage between the course content, the learning activity (practice quiz), and the summative assessment test. True/false questions were replaced with negatively framed single best answer (SBA) questions. The underlying rationales were that we wanted to expose the students to a broad range of correct statements about pharmacology, and that real-life situations require the ability to recognize aberrant ideas among a set of plausible alternatives. In step II, as in step I, the practice quizzes were available throughout the course and the summative test was available during the final 2 weeks of the course and unlimited attempts were allowed. Each time the students received nine randomly selected SBA questions drawn from 37 (psychiatry) and 36 (neurology) questions. A 100% score was required to pass the test.

### Evaluation

Before and after each quiz implementation step, the students’ achieved level of post-course knowledge was investigated through an anonymous and voluntary, paper-based knowledge evaluation test. This consisted of 20 case-based SBA questions with four response alternatives (Additional file 2, Questionnaire). The SBA questions did not map directly to the practice quizzes and the summative assessment test, but concerned the application of pharmacological knowledge in a clinical context. In addition, to inform future development of learning activities in the courses, as well as the design of practice quizzes and summative tests, a questionnaire was administered including questions asking students to rate statements on their confidence in various aspects of prescribing using a 5-point Likert scale (1 = totally disagree to 5 = totally agree). In two open-ended questions, students were invited to comment on helpful educational components and suggest improvements for developing their prescribing skills for their future professional life. Basic demographic and course-related information was also collected. In step II, two additional questions were asked regarding how extensively the students had used practice quizzes and how many times they had used the summative assessment test.

### Participants and data collection

A total of 404 medical students were enrolled in the two courses across the three course instances during which the study was conducted: 124 at baseline (autumn 2022), 140 at step I (spring 2023), and 140 at step II (autumn 2023). At the beginning of their Psychiatry or Neurology course, the students received oral information about the study. All were invited to participate. The only inclusion criterion was enrollment in one of the courses, and no exclusion criteria were applied. Written information was provided on the learning platform, including that the voluntary knowledge evaluation test/questionnaire was expected to take a little less than 1 h to complete. Participation was voluntary, and the students were informed that neither participation nor test results would affect course assessments.

After completing their Psychiatry or Neurology course, the students were invited to take the knowledge evaluation test and complete the questionnaire at a scheduled session adjacent to a mandatory learning activity during the first week of the next course. Printed copies were provided and by submitting a completed test/questionnaire, the students gave informed consent to participate. Students could optionally mark their test with a self-generated code. These codes, along with corresponding scores, were later published on the learning platform, allowing students to view their results while maintaining anonymity. The students were compensated for their participation with a sandwich lunch and two lottery tickets.

### Analysis

The statistical analyses were performed using SPSS (IBM SPSS Statistics for Windows, version 27.0; IBM Corp., Armonk, NY, USA). The sample size was based on available students in the courses involved; no power analysis was made beforehand. Mann–Whitney and chi-square tests were used for comparisons between students participating in courses before and after the implementation of autograded practice quizzes and summative assessment tests. For dichotomized analyses of the knowledge evaluation test results, cut-off scores were 65% and 85% of correct answers. The 65% threshold was based on the pass range (57–73%) of the United Kingdom Prescribing Safety Assessment [9], while 85% is the pass mark used in the Dutch National Pharmacotherapy Assessment [10]. Correlation coefficients (r) were calculated using Spearman rank correlation. Descriptive results are reported as numbers (percentages) or medians (lower to upper quartile). Where relevant, the mean ± standard deviation is provided to facilitate interpretation. For dichotomized analyses of the questionnaire results, ratings of 4 or 5 were classified as agreement with the statement.

The free-text replies were analysed using manifest content analysis [11] guided by the research question “What in their education do medical students consider important in enabling them to treat patients with medications?” All authors contributed to the qualitative analysis, based on their diverse professional perspectives, from that of being a physician specialized in clinical pharmacology and a Professor of Pharmacotherapy (SMW); to that of a physician specialized in neurology and an Associate Professor (KJ) or Professor of Neurology (JZ, FB); or of a physician specialized in psychiatry and either an Associate Professor (SS) or a Professor of Psychiatry (EW). In addition, the authors were involved in the Basic Pharmacology course (SMW, FB) or were course leaders/examiners in the Neurology (KJ, JZ) or Psychiatry (SS, EW) course. All authors have broad experience in teaching.

In the first step of the qualitative analysis, three author pairs independently identified, extracted and coded meaning units, followed by a consensus discussion (baseline: KJ and JZ; step I: SS and EW; step II: SMW and FB). As the analysis progressed, similar codes kept reappearing, while no new codes emerged, indicating data saturation. NVivo 14 (QSR International, Melbourne, Australia) was used to manage data from step II. Next, all authors discussed the extractions of step II codes and performed a data-driven inductive thematic analysis without any predetermined categories or themes. This involved repeatedly sorting the various codes into categories until consensus was reached. Emergent themes were identified as overarching interpretative concepts. Finally, the meaning units identified at baseline and during step I were again reviewed to ensure that no categories or themes had been overlooked.

## Results

In all, 274 students participated in the study across the three course instances (response rate: 68%; baseline: *n* = 85, 69%; step I: *n* = 96, 69%; step II: *n* = 93, 66%). Overall, 152 (56%) were women and 180 (66%) were 24 years or younger (Table 1). A total of 114 students (57%) filled out the paper-based knowledge evaluation test/questionnaire after completing the Psychiatry course, and 160 (77%) did so after completing the Neurology course. All students who participated in steps I and II reported that they had passed the summative assessment test.

**Table 1:** Characteristics of the participants at each of the three course instances included in the present study: at baseline (autumn 2022) and after steps I (spring 2023) and II (autumn 2023) of the implementations of autograded practice quizzes and summative assessment tests. Values are presented as n (%)

		Baseline *n* = 85	Step I *n* = 96	Step II *n* = 93
Gender	Women	48 (56)	51 (53)	53 (57)
	Men	35 (41)	42 (44)	39 (42)
	Other/NR	2 (2)	3 (3)	1 (1)
Age	≤24 years	54 (64)	49 (51)	77 (83)
	> 24 years	28 (33)	44 (46)	15 (16)
	NR	3 (4)	3 (3)	1 (1)
PhD degree	No	77 (91)	92 (96)	92 (99)
	Yes	2 (2)	3 (3)	1 (1)
	NR	6 (7)	1 (1)	0
Knowledge test performed	Psychiatry	33 (39)	42 (44)	39 (42)
	Neurology	52 (61)	54 (56)	54 (58)

*NR* not reported

### Quantitative analysis

Before the implementation of quizzes, the median of correct answers in the knowledge evaluation test was 10 (9‒13), with 24 students (28%) achieving ≥65% in the test (a score of ≥ 13, see Table 2). After step I, the students scored 11 (8–13) correct answers (*P* = 0.88 versus baseline), and 31 (32%) had ≥65% correct answers (*P* = 0.55 versus baseline). Students taking the test after step II got 14 (12–16) answers correct; 64 (69%) had ≥65% correct answers (*P* < 0.0001 versus both baseline and step I).

**Table 2:** Results of the knowledge evaluation test (maximum score: 20 correct answers) voluntarily completed by students during the week following each of the three course instances included in the present study: at baseline (autumn 2022) and after steps I (spring 2023) and II (autumn 2023) of the implementation of autograded practice quizzes and summative assessment tests. Statistically significant differences are bolded

	Correct answers	Baseline *n* = 85	Step I^a^ *n* = 96	Step II^b^ *n* = 93	*P* value step II vs. baseline/ step II vs. step I
Total	Median (Q1‒Q3)	10 (9‒13)	11 (8‒13)	14 (12‒16)	**< 0.0001/<0.0001**
	≥ 65%, n (%)^c^	24 (28)	31 (32)	64 (69)	**< 0.0001/<0.0001**
	≥ 85%, n (%)^d^	1 (1)	5 (5)	19 (20)	**< 0.0001/0.002**
Psychiatry	Median (Q1‒Q3)	12 (10‒15)	13 (11‒14)	16 (15‒18)	**< 0.0001//<0.0001**
	≥ 65%, n (%)^c^	16 (48)	23 (55)	36 (92)	**< 0.0001/0.0001**
	≥ 85%, n (%)^d^	1 (3)	5 (12)	17 (44)	**0.0001/0.001**
Neurology	Median (Q1‒Q3)	9.5 (8‒12)	9.5 (8‒11)	13 (10‒15)	**0.0001/<0.0001**
	≥ 65%, n (%)^c^	8 (15)	8 (15)	28 (52)	**0.0001/<0.0001**
	≥ 85%, n (%)^d^	0	0	2 (4)	0.16/0.15

*NA* not applicable, *Q1* lower quartile, *Q3* upper quartile

^a^Practice and summative assessment quizzes; the assessment quiz was open during the last 2 weeks of the course and included a random selection of ten true/false statements at every attempt (no restriction of attempts) out of a pool of 265 (psychiatry) and 157 (neurology) statements; eight correct answers were required to pass

^b^Practice and summative assessment quizzes; the assessment quiz was open during the last 2 weeks of the course and included a random selection of nine single best answer (SBA) questions at every attempt (no restriction of attempts) out of a pool of 37 (psychiatry) and 36 (neurology) SBA questions; nine correct answers were required to pass. The SBA questions were elaborated with details of clinical contexts according to the World Health Organization (WHO) six-step model for good prescribing [8], and there was an increased focus on constructive alignment

^c^The cut-off of 65% was chosen as it lies within the United Kingdom Prescribing Safety Assessment pass range of 57–73% [9]

^d^The cut-off of 85% was chosen based on the Dutch National Pharmacotherapy Assessment pass mark of 85% [10]

In total, 109 students (58%) reported having used the practice quizzes implemented in steps I and II (Table 3). After step II, the extent of practice quiz use was positively correlated with scores in the knowledge evaluation test (*r* = 0.33; *P* = 0.002). The median number of student-reported attempts on the summative test after step II was 6 (3–9). There was a negative correlation between the number of summative test attempts and both the extent of practice quiz use (*r*=–0.22; *P* = 0.034) and the number of correct answers on the knowledge evaluation test (*r*=–0.44; *P* < 0.0001).

**Table 3:** Use of autograded practice quizzes and summative assessment tests on the learning platform regarding pharmacotherapeutic knowledge, in each of the two course instances in which they were implemented: step I (spring 2023) and step II (autumn 2023)

		Step I *n* = 96	Step II *n* = 93
Used the practice quizzes, n (%)	Yes	54 (56)	55 (59)
	No	42 (44)	37 (40)
	NR	0	1 (1)
Extent of practice-quiz use^a^, n (%)	Used all	NA	16 (17)
	Used most	NA	20 (22)
	Used some	NA	29 (31)
	Did not use any	NA	27 (29)
	NR	NA	1 (1)
Performed the summative assessment test, reported number of times^b^	Median (Q1‒Q3)	NA	6 (3‒9)
	Range	NA	1‒25

*NA* not applicable, *NR* not reported, *Q1* lower quartile, *Q3* upper quartile

^a^This question was not included in the step I questionnaire. In step II, one student did not respond to it. The response alternatives were presented as shown, and did not specify the number of quizzes used

^b^This question was not included in the step I questionnaire. Passing this test was required to pass the course

Regarding confidence in various aspects related to prescribing, 75% of the entire cohort (*n* = 274) reported that they felt confident in writing medication discharge summaries while 43% reported confidence in performing medication reviews (Table 4). Familiarity with the procedures and systems of writing prescriptions was reported by 23%, while 42% were familiar with the corresponding procedures for prescribing to inpatients. In terms of pharmacological knowledge, 20% expressed confidence in knowing the mechanisms of action, effects and side effects of common medications. 9% felt confident about dosages, and 15% about pharmacokinetic principles. Regarding other aspects of prescribing, 36% stated that they had sufficient knowledge to evaluate clinical trials, 28% reported sufficient knowledge of regulations related to medications, and 24% thought that the medical programme “so far had prepared them well” for prescribing in their future clinical practice. Comparing student responses after the step II implementation with baseline, there were significant differences in how confident they felt about dosages (4% versus 13%, *P* = 0.038) and in their perception that the medical program “so far had prepared them well” for prescribing (19% versus 35%, *P* = 0.014).

**Table 4:** Student agreement with ten statements related to prescribing knowledge and skills following each of the three course instances included in the present study: at baseline (autumn 2022) and after steps I (spring 2023) and II (autumn 2023) of the quiz implementation. Responses are presented as the Likert scale (1 = totally disagree and 5 = totally agree) median (interquartile range) and, to facilitate interpretation, also as mean±standard deviation in italics. Students that agreed (responded with a 4 or 5 on the Likert scale) are reported as numbers (percentages). Statistically significant differences are bolded

		Baseline *n* = 85	Step I *n* = 96	Step II *n* = 93	*P* value Step II vs. baseline/ Step II vs. step I
1. I feel confident in my knowledge of common psychiatric/neurological medications regarding mechanisms of action, effects, and side effects.	Response	3 (2‒3) *2.8 ± 0.9*	3 (2‒3) *2.5 ± 1.0*	3 (2‒3) *2.8 ± 0.9*	0.95/**0.025**
	Agree	18 (21)	15 (16)	22 (24)	0.69/0.16
2. I feel confident in my knowledge of common psychiatric/neurological medications regarding dosage	Response	2 (1.5‒2.5) *2.2 ± 1.0*	2 (1‒3) *2.2 ± 0.9*	2 (1‒2) *2.0 ± 0.8*	0.58/0.12
	Agree	11 (13)	9 (9)	4 (4)	**0.038**/0.16
3. I feel confident in my knowledge of the principles of pharmacokinetics related to common psychiatric/neurological medications.	Response	2 (2‒3) *2.4 ± 1.0*	2 (2‒3) *2.4 ± 1.0*	2 (2‒3) *2.5 ± 0.9*	0.35/0.26
	Agree	14 (16)	14 (15)	13 (14)	0.64/0.91
4. I feel confident in performing medication reviews.	Response	3 (2‒4) *3.2 ± 1.2*	3 (2‒4) *3.1 ± 1.1*	3 (2‒4) *3.3 ± 1.0*	0.96/0.22
	Agree	42 (49)	34 (35)	42 (45)	0.57/0.19
5. I feel confident in writing medication discharge summaries.	Response	4 (4‒5) *4.2 ± 1.0*	4 (3‒5) *3.9 ± 1.1*	4 (3‒5) *4.0 ± 0.9*	0.14/0.69
	Agree	68 (80)	70 (73)	68 (73)	0.28/0.98
6. I am familiar with the procedures/systems of writing prescriptions for medications.	Response	2 (1.5‒4) *2.6 ± 1.3*	2 (1‒3) *2.2 ± 1.3*	2 (1‒4) *2.4 ± 1.3*	0.31/0.42
	Agree	22 (26)	18 (19)	24 (26)	0.99/0.26
7. I am familiar with the procedures/systems of prescribing medications to inpatients	Response	3 (2‒4) *3.2 ± 1.4*	3 (2‒4) *2.7 ± 1.3*	3 (2‒4.5) *3.2 ± 1.5*	0.84/**0.027**
	Agree	38 (45)	32 (33)	46 (49)	0.53/**0.024**
8. I have sufficient knowledge to evaluate clinical trials, for example, to avoid being misled by marketing.	Response	3 (2‒4) *3.0 ± 1.1*	3 (2‒4) *2.8 ± 1.1*	3 (2.5‒4) *3.2 ± 1.0*	0.22/**0.010**
	Agree	29 (34)	27 (28)	42 (45)	0.13/**0.015**
9. I have sufficient knowledge of overarching regulations related to medications, such as the Pharmaceutical Benefit System.	Response	3 (2‒4) *2.8 ± 1.1*	3 (2‒4) *2.9 ± 1.0*	3 (2‒4) *2.7 ± 1.0*	0.60/0.22
	Agree	23 (27)	31 (32)	24 (26)	0.92/0.37
10. I think that the medical program has so far prepared me well for my role as a doctor when it comes to treating patients with medications.	Response	3 (2‒4) *3.0 ± 1.1*	3.(2‒3) *2.6 ± 1.0*	3 (2‒3) *2.7 ± 0.9*	0.058/0.58
	Agree	30 (35)	18 (19)	18 (19)	**0.014/**0.92

In response to the open-ended question regarding helpful components for professional development in terms of the physician’s responsibility for patients’ pharmacotherapy, 171 students (62%) mentioned clinical placements. Among the 114 students who responded after the Psychiatry course, where the ward-based education constitutes a larger part of the course compared with the Neurology course, 92 (81%) mentioned clinical placements.

### Qualitative analysis

**Fig. 1:**
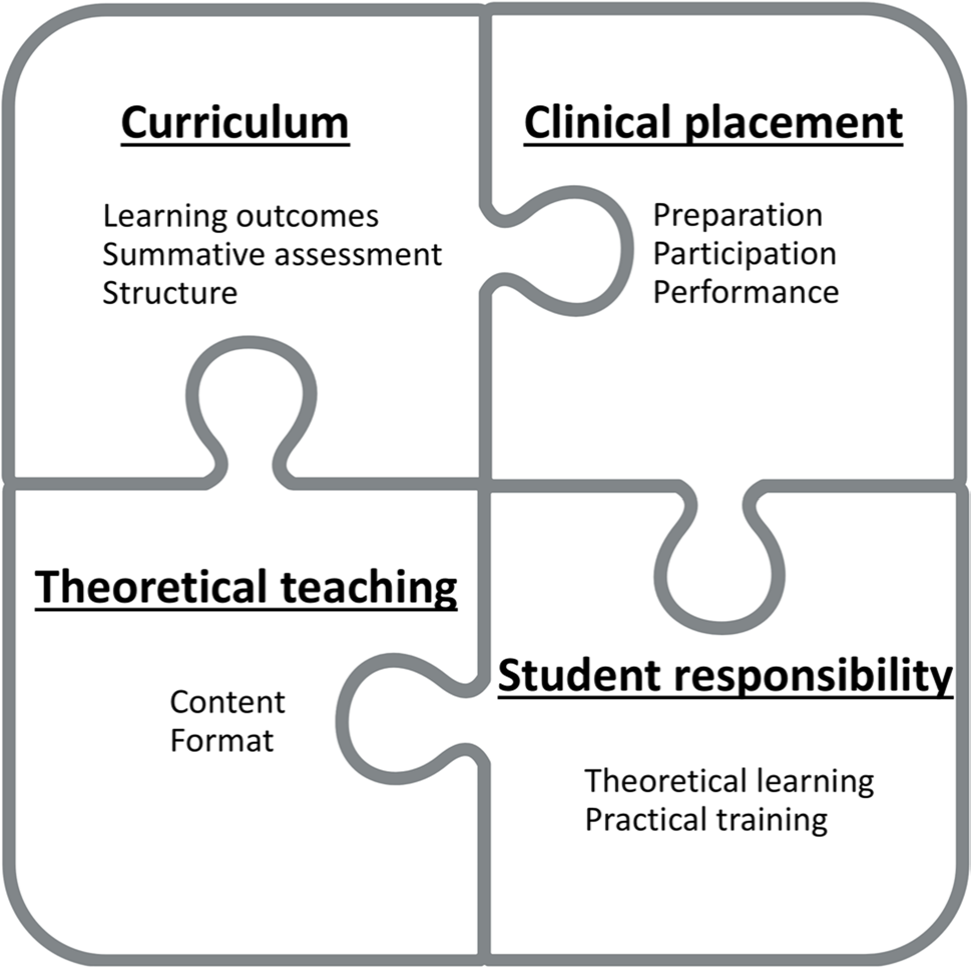
Themes (underscored, bold type) and categories that emerged from the meaning units identified in the content analysis guided by the research question “What in their education do medical students consider important in enabling them to treat patients with medications?”

All participants provided free text responses to the open-ended questions. Four themes emerged from the content analysis: *Curriculum*, *Clinical placement*, *Theoretical teaching* and *Student responsibility* (Fig. 1). The themes and categories within the themes are described below, along with example quotes after the second step of the implementation. Answers to the question “Elements that were helpful…” are referred to as “helpful components (H)”, and answers to the question “To prepare me better…” as “improvement potential (I)”. The letter “P” indicates responses after the Psychiatry course, and “N” after the Neurology course. In Additional file 1 (Table S2), categories and codes forming the basis for the emerging themes are presented along with more example quotes, from before and after quiz implementation.

The theme *Curriculum* emerged from three categories: *Learning outcomes*, *Structure* and *Summative assessment*. This theme illustrates the teacher’s responsibility to clearly define sufficiently demanding learning outcomes; a thought-through structure of the education including learning activities, repetition and progress; and the need for summative assessments. The following example quotes illustrate components within this theme: “*There were higher demands on knowledge about medications*,* like in this course for instance*” (*Learning outcomes*, H, N); “*More repetition during the education. Easy to forget medications even from just 6 months ago*” (*Structure*, I, P); and “*The summative quiz was also a good resource. This was a good way to test yourself as well*” (*Summative assessments*, H, P).

The theme *Clinical placement* included categories concerning the translation of theoretical knowledge into medical practice. The category *Preparation* focused on the systems used and the decision support available; *Participation* included listening to and discussing pharmacotherapeutic reasoning with physicians; and *Performance* involved the training needed over the entire prescribing process including medication reviews. The following example quotes illustrate components within this theme: “[If] *I could write prescriptions*,* prescribe medications*,* inject medications etc. under supervision during clinical placements*” (*Preparation*, I, N); “*My supervisors at the clinical placement who asked a lot of questions and involved us students in the medication-related work tasks*” (*Participation*, H, P); and “*It would have been helpful to do everything myself from the start. Like: here is a patient*,* these are the symptoms. Which medications? Where do you click in Melior* [the electronic medical record system]*? Talk to the patient. All steps of the way*” (*Performance*, I, P).

The theme *Theoretical teaching* included the two categories *Content* and *Format*, reflecting students’ preferences as to how the Pharmacology and Pharmacotherapy syllabus should be taught as well as useful and varying pedagogical models including linkage to clinical practice. The following example quotes illustrate components within this theme: “*There was more clarity about pharmacotherapy being fundamental* [to the medical profession]” (*Content*, I, N); and “*I enjoyed the medication quizzes and wish every clinical course included these elements. It feels like you learn in a more structured way then and that you don’t forget to read about medications in FASS* [Pharmaceutical Specialities in Sweden]” (*Format*, I, P).

*Student responsibility* emerged as a theme on its own as it centres on the student’s role in the learning process, in contrast to the other three themes which primarily reflect teacher-driven elements. The following example quote illustrates the two categories within this theme, *Theoretical learning* and *Practical learning*: “… *I did the medication quizzes and more medication reviews at the clinical placements*” (I, N).

## Discussion

The quantitative part of this mixed methods study shows that the pharmacotherapeutic knowledge among medical students can be substantially increased by using autograded practice and assessment quizzes about medications on the digital learning platform during clinical courses, elaborated with details of clinical contexts and constructive alignment, and with high criteria for passing. The qualitative findings complement these results by identifying four emerging themes reflecting educational components that medical students find important for learning how to treat patients with medications: curriculum structure, clinical placements, theoretical teaching, and student responsibility.

Given the complexity of prescribing―differential diagnosis, medication selection, communication, and documentation [8] ―it is not surprising that embedding pharmacotherapy within clinical contexts is critical for learning. Indeed, the value of clinical placement has been emphasized previously [5]. Furthermore, from a constructivist perspective, the clinical context enables learning by allowing students to construct meaning as they interpret real-world information within their existing cognitive frameworks; their experiences are repeatedly processed through prior knowledge and integrated into the development of new understanding [12]. Our quantitative findings―that integrated clinical contexts in the pharmacotherapeutic quizzes in step II of the implementation were required for positive learning effects―are in line with this reasoning. Previous studies, based on students’ reported perceptions, further support the value of clinical placement as this provides opportunities to conduct medication reviews and write medication discharge summaries [13, 14], two components highlighted also in the current qualitative analysis.

The qualitative results provide additional and nuanced insights about clinical placement as an essential educational setting for medical students. The theme *Clinical placements* contains three categories reflecting components of gradually increased independence. First, the students must be prepared for medication-related patient work, for instance by being introduced to the electronic prescribing system and decision supports available. Next, they benefit from actively participating in medication-related professional work, hearing about and discussing pharmacotherapy with supervising physicians. Finally, they must perform work themselves, including the entire process as outlined in the WHO practical manual to good prescribing [8]. Notably in our study, few students were familiar with the procedures and systems for writing prescriptions. Since the current electronic medical record system does not permit students to prescribe under supervision, this gap points to the need for better integration between medical education and healthcare. Educational priorities should be considered in system design as well as in procurement. Indeed, supervised preprescribing (and its accompanying formative assessment) using hospital inpatient electronic prescribing systems has been found to benefit student learning [15].

The qualitative findings also support that students value teaching that is closely linked to clinical practice. Indeed, the students preferred theoretical teaching to be linked to patients and diseases, case scenarios and simulations. In the development of quizzes in the current study, we had the advantage of all being physicians, the majority with vast clinical experience. Such backgrounds facilitate relevant linkage to clinical practice in teaching, also when basic pharmacology―including pharmacodynamics and pharmacokinetics―is taught via quizzes. In a previous study, students attending a medical programme that introduced problem-based learning in pharmacology and clinical pharmacology achieved higher knowledge scores in a prescribing assessment, compared with students attending a programme based on traditional lectures [16]. Furthermore, problem-based learning has been described as beneficial in pharmacology [17]. While separate prescribing assessments have been conducted in some European countries with positive experiences [18, 19], our results suggest that structured integration of pharmacology and pharmacotherapy in clinical courses can be a feasible alternative. Such integration allows students to begin forming therapy “scripts”, which are vital for clinical and therapeutic reasoning [20].

From a teacher’s perspective, a well-designed curriculum is key to facilitating medical students’ attainment of sufficient skills to hold a prescribing licence. Our quantitative findings underscore the importance of constructive alignment, ensuring that learning outcomes, activities and assessments are mutually reinforcing [21]. Only after such alignment was achieved did the performance on the knowledge evaluation test improve. Furthermore, the qualitative results provide concrete information regarding important educational components worthy of consideration. One example is that the students requested higher levels of medication-related learning objectives and more rigorous summative assessments, reflecting their appreciation of the importance of pharmacotherapy.

Interestingly, the students expressed that they would like clearer communication about the importance of pharmacotherapy in their future professional role as a physician. Although it can only be speculated whether our pharmacotherapeutic quizzes contribute to such understanding, one possible interpretation of the students’ lower agreement with statements about feeling sufficiently prepared after the quiz implementation is that this reflects an increased awareness of the importance of pharmacotherapy. Nevertheless, the questionnaire responses suggest that the physician’s role in pharmacotherapy is often unclear to medical students, potentially reflecting a lack of explicit emphasis on pharmacotherapy in the national learning objectives. In fact, although most of Sweden’s 23 national medical education outcomes include pharmacotherapy-related elements, the term itself is not explicitly mentioned [22]. Similarly, the recently introduced Entrustable Professional Activities (EPAs) all include medication-related aspects, but pharmacotherapy is rarely explicitly mentioned [23]. It is possible that if the licence to prescribe is not explicitly discussed, students may not readily perceive prescribing as a professional responsibility. Therefore, teachers involved in medical curriculum development must ensure that pharmacotherapy is clearly represented in learning outcomes and teaching practice [24]. Furthermore, the students’ appreciation of having shortlists of medications in focus illustrates a need for clear guidance and limits for the contents of the clinical courses. Guidance on medication classes with which graduates should be familiar has recently been updated by the British Pharmacological Society [25].

After implementation of step II, the median self-reported number of performed summative assessment tests was six. After step I, the median number of attempts needed to pass was two among all course participants, according to data from the digital learning platform (data not shown). Consequently, as negative SBA questions were used with only one incorrect statement, the median student was exposed to 162 correct statements in step II. In step I, the median student was exposed to only 20 simple true/false statements and had to correctly identify only 16 of these. The increased requirements for passing, as well as the increased exposure to correct statements in step II may have benefited learning. Furthermore, our results are in line with a review of assessment methods för prescribing competence, which listed true-false questions as having a very poor impact on learning [26].

Interestingly, students who engaged more with practice quizzes required fewer summative test attempts and performed better on the knowledge evaluation test. Thus, the quiz design allowed students to choose their learning strategy―frequent practice or repeated assessment―while still achieving educational objectives. Students’ free-text responses further emphasized the value of practice quizzes, with some students suggesting that they should be mandatory. The quizzes also prepared for use of real-world decision support tools during clinical placements. Conversely, students who needed multiple summative test attempts tended to perform worse in the knowledge evaluation test, highlighting the effectiveness of active engagement through practice quizzes. These results are in line with previous research on quizzes as a learning strategy [6, 27, 28].

Digital education offers the advantage of scaling. First, because the quizzes were autograded, the students automatically received computer-assisted instructional feedback, which has been described as beneficial for learning [29]. Second, once developed, it can be transferred from one course to another without much teacher effort, particularly in this case where the content is largely based on overall stable SPCs. Consequently, our findings, that the elaborated quizzes were favourable for student learning, support further use. However, not all students performed well in the knowledge evaluation test, and many still reported low confidence in core pharmacological knowledge. This underscores that quizzes alone are not a comprehensive solution. Additional strategies―such as more salient pharmacotherapy objectives, dedicated course time, and enhanced supervision during clinical placements―are needed. These strategies also align with previous quantitative and qualitative results. Time specifically allocated to pharmacotherapy-related education including collegial discussions is essential for junior doctors before acquiring the prescribing licence [30, 31].

The emergence of learning as a student responsibility, as a theme on its own, is encouraging. The formative aspect of the knowledge evaluation test may have contributed to students’ recognition of their learning needs. Indeed, prior research has indicated that formative tests can enhance students’ learning outcomes [32]. Critical reasoning exercises, as part of a formative assessment process, may also stimulate self-directed learning skills [33]. It must be noted, however, that when a pharmacotherapeutic assessment is limited to being formative and not summative, medical students score significantly lower [34].

An important strength of the current study is that it describes in detail an educational intervention that increases medical students’ pharmacotherapeutic knowledge. This knowledge is required in patient care, and is an essential clinical competency for future physicians responsible for patients’ pharmacotherapy. Other initiatives aimed at improved prescribing practices have often involved other professions. The three, to our knowledge, largest randomized controlled trials on the topic have, however, failed to show patient benefits of such interventions [35–37]. We have by contrast focused the educational intervention on medical students, i.e., future professionals who will eventually be responsible for prescribing. Given both the potential benefit of good prescribing and the harms of suboptimal prescribing―including adverse drug reactions and unnecessary healthcare utilization―this is a critical area for investment. It should be noted as a limitation of this study, however, that we were unable to link the practice-quiz use and summative assessment test results to the overall knowledge evaluation tests, as the latter were anonymous. Potential associations in this regard may be worth investigating in future studies. Furthermore, comparing final course exam results before and after the implementation of the digital quizzes for learning and assessment was not meaningful; pharmacotherapeutic questions constitute only a minority of the items in these exams and students’ performance may vary from one semester to another.

We have previously reported that students who choose to participate in education-related research may perform better than those who do not [38], and this must be considered in the interpretation of the overall achieved level of knowledge in the present study. Furthermore, the single-site setting is a limitation with respect to the generalizability of the results. Conversely, the high participation rate in this study can be considered a strength in terms of generalizability. Another strength is that the quizzes were implemented in parallel across two separate courses. With similar findings in both, showing clear benefits of implementing pharmacotherapeutic quizzes that provide contextual details and constructive alignment, the benefits of this educational intervention appear robust. Although the non-randomized design has implications for the certainty of evidence, the consistency in course pass rates (92% at baseline and 95% in steps I and II) suggests that differences in student cohorts are unlikely to explain the observed effects.

## Conclusions

Autograded practice and assessment quizzes enhanced by providing clinical contexts and aligned with learning objectives seem to effectively increase pharmacotherapeutic knowledge among medical students. Once implemented, limited teacher input is needed for maintenance of these digital learning tools. Moving forward, further course developments related to medical students’ acquisition of pharmacotherapeutic knowledge and skills could focus on the four emergent themes regarding what students consider important: curriculum structure, clinical placements, theoretical teaching, and student responsibility. Of these, clinical placements deserve particular emphasis from an educational perspective. Teachers could focus on promoting students’ preparation for, and participation and performance in, this clinical educational setting.

## Supplementary Information

Additional file 1: Table S1 Description of practice quizzes and summative assessment tests. Table S2 Example quotes for the themes, categories and codes that emerged in the qualitative analysis.

Additional file 2: Questionnaire.

## Data Availability

The datasets analysed during the current study are available from the corresponding author upon reasonable request.
