# Supplementary material for: Helpful if “Medication took up more space in the course!” – a mixed methods study about pharmacotherapeutic knowledge and digital quizzes for learning and assessment in the medical programme
Source: BMC Med Educ. 2026 Apr 30;26:706. doi: 10.1186/s12909-026-09292-7 (PMC13130682; doi:10.1186/s12909-026-09292-7)
Supplement: Supplementary file 1 — Additional file 1: Table S1 Description of practice quizzes and summative assessment tests. Table S2 Example quotes for the themes, categories and codes that emerged in the qualitative analysis. [file 12909_2026_9292_MOESM1_ESM.docx]

**Table S1** Description of digital practice quizzes and digital summative assessment tests during step I (spring 2023) and step II (autumn 2023) of the implementation. This table is also included in an article in Swedish.^1^

|  |  | | **Step I** | **Step II** |
| --- | --- | --- | --- | --- |
| Practice quizzes | Medicinal drugs in focus, n | | 46 (psychiatry)  33 (neurology) | 46 (psychiatry)  42 (neurology) |
|  | Medication groups/therapeutic areas | | Antidepressants, anxiolytics, hypnotics, medications used in addiction, antipsychotics, mood stabilizers, other medications including central stimulants (psychiatry)  Medications used in (i) stroke, (ii) epilepsy, (iii) headache, (iv) neurodegenerative disease, tremor, and Willis-Ekbom disease; and (v) other medications including immunomodulating agents (neurology) | |
|  | Structure | | Eight quizzes: one per medication group and one containing a random sample drawn from the full question pool; 56 questions in total, with 3–18 questions per quiz (psychiatry)  One quiz with a random selection of six questions (drawn from a pool of 47 questions) for each attempt (neurology) | Seven quizzes: one per medication group; 58 questions in total, with 4–19 questions per quiz (psychiatry)  Six quizzes: one per therapeutic area and one covering all therapeutic areas, each presenting a random selection of six questions (drawn from a pool of 47 questions) for each attempt (neurology) |
|  | Clinical context | | - | According to the WHO 6-step model |
|  | 1 example question per course and development step | | Psychiatry:  Match each substance to its correct mechanism of action  (8 substances, 5 drop-down answers)  Neurology:  After an ischemic stroke without a cardiac source of embolism, antiplatelet therapy is recommended as secondary prevention. Treatment with low‑dose acetylsalicylic acid can reduce the risk of a new ischemic stroke by approximately 25%, and the effect of clopidogrel is of roughly the same magnitude. Indicate whether the statements below are true or false.  (6 statements, true/false drop-down answers) | Psychiatry:  Why do you need to understand the mechanisms of action of anxiolytic medications? Description according to the WHO 6-step model:  *WHO step 3* (Assess whether the recommended first‑line treatment is appropriate for the patient; individualize treatment): Based on the patient’s individual circumstances, the mechanism of action of the medication may be crucial for selecting the most suitable treatment. For anxiolytic drugs that act on the GABA system, for example, there is a risk of developing dependence. These medications may therefore be advisable to avoid in patients with an existing or increased risk of substance dependence.  *WHO step 5* (Inform and instruct the patient): The mechanism of action can help guide which anxiolytics/ataractics should not be combined with alcohol. Benzodiazepines enhance GABA transmission (via GABA‑A receptors), and alcohol also stimulates GABA‑A receptors (which become downregulated during long‑term alcohol use). Therefore, alcohol should be avoided during treatment with benzodiazepines.  Match each substance to its correct mechanism of action  (8 substances, 5 drop-down answers)  Neurology:  After an ischemic stroke without a cardiac source of embolism, antiplatelet therapy is recommended as secondary prevention. Treatment with low‑dose acetylsalicylic acid can reduce the risk of a new ischemic stroke by approximately 25%, and the effect of clopidogrel is of roughly the same magnitude. Indicate whether the statements below are true or false.  (WHO Step 2 – Treatment goals, Step 3 – Individualized treatment)  (6 statements, true/false drop-down answers) |
|  | Links within the questions to resources for drug information and medical decision support | | Pharmaceutical Specialities in Sweden (FASS) | Pharmaceutical Specialities in Sweden (FASS)  CDSS (Janusmed^2^)  Regional list of recommended medications (REKlistan) |
|  | Other minor revisions | Integration in clinical placement | - | Quick reference guide regarding the six steps in the WHO *Guide to good prescribing* was distributed to supervisors in clinical placement |
|  |  | Short films on the learning platform | - | Introduction to pharmacotherapy in psychiatry/neurology  Drug interactions: repetition and a quick guide to the CDSS (Janusmed^2^) |
| Summative assessment tests | Questions, n | | 10 | 9 |
|  | Structure | | Random sample from a question pool including 265 (psychiatry)/157 (neurology) true/false statements | Random sample from a question pool including 37 (psychiatry)/36 (neurology) single best answer questions structured in groups according to the WHO 6-step model, with an introductory clinical case context |
|  | 1 example question per course and development step | | Psychiatry:  Based on the pharmacological properties of haloperidol, it can be expected that patients quite often experience a dry mouth as an adverse drug reaction (true/false?)  Neurology:  A reasonable oral loading dose of acetylsalicylic acid after a transient ischemic attach is 300 mg (true/false?) | Psychiatry, WHO step 1: *Define the patient’s problem (differential diagnosis)*:  You are doing rounds in the ward during a temporary position at a psychiatric clinic. The nurse reports about a patient who has been anxious and shaky in the early morning, shows abnormal test results for another patient, and wants you to assess a male patient who is worried about changes in his right breast. Below are four statements related to the pharmacotherapy and differential diagnostic considerations that may be relevant. Which statement is LEAST reasonable?   - If a patient has missed a single dose of fluoxetine, this can explain withdrawal symptoms as this medication’s short half-life contributes to this type of issue. - For a patient being treated with paliperidone and showing gynecomastia, there is good reason to suspect a medication side effect – this medication is associated with increased prolactin levels. - If a patient being treated with lithium shows rapid hand tremor, there is good reason to suspect a medication side effect. - In the case of hyponatremia during ongoing treatment with fluoxetine, there is reason to suspect a medication side effect, especially if the patient is also on bendroflumethiazide.   Neurology, WHO step 3: *Assess if the recommended first-line treatment is appropriate for the patient, and individualize it*:  A 34-year-old woman who works as a chief executive officer consults you due to hand tremors. The symptoms have been present for over 10 years and have slowly worsened. She is now starting to avoid certain tasks at work because she feels stared at. She recognizes the symptoms as essential tremor, the same condition her father has had for many years, as well as others in his family. She asks for some treatment to take as needed for the tremors. Which statement is LEAST reasonable?   - Salbutamol is a reasonable second-line treatment for essential tremor. - If you plan to prescribe propranolol as needed to a patient where you have just diagnosed essential tremor, you should investigate any asthmatic issues first. - A substance that can exacerbate essential tremor is nicotine, so the patient can be advised to avoid it. - If you plan to prescribe propranolol as needed to a young patient where you have just diagnosed essential tremor, it is good to inform them that it limits performance during exercise |
|  | Time limit | | 5 minutes | 10 minutes (psychiatry)  15 minutes (neurology) |
|  | Attempt limit | | No limit, the best result counts | No limit, the best result counts |
|  | Required to pass | | 8 correct answers | 9 correct answers |
|  | Constructive alignment | | Yes | Enhanced^3^ |

CDSS = clinical decision support system, WHO = World Health Organization

^1^Wallerstedt SM, Jood K, Kern S, Rönnbäck C, Steingrimsson S, Wentz E, Zelano J, Bergquist F. [Webb-baserade quiz för övning och examination avseende kunskap om läkemedelsbehandling under kliniska kurser – en pilotstudie på psykiatri- och neurologikurserna]. Läkartidningen 2024;121:23212

^2^Drug interactions, risk profile, fetal effects, kidney function

^3^In step II, the students were exposed to a larger portion of the medication-related course content during the quiz, i.e., an increased linkage between course content, learning activity (practice quiz), and summative assessment test

**Table S2** Example quotes for the themes, categories, and codes that emerged in the qualitative analysis

| **Theme** | **Category** | **Code** | **Example quotes** | |
| --- | --- | --- | --- | --- |
|  |  |  | **Before/step I*** | **Step II*** |
| Curriculum | Learning outcomes | Higher level of medication-related learning objectives | “Higher requirements regarding medication knowledge” (I, N139) | “There were higher demands on knowledge about medications, like in this course for instance.” (I, N202) |
|  |  | Clearly stated medication-related learning objectives | “List of medications at the beginning of the course including mechanism of action and indication, pre-treatment lab tests and "NOTE" information. This would have helped to know what to focus on later during the course.” (I, P7) | “That we were focused early [in the course] on 46 selected medications” (H, P227) |
|  |  | More focus on medications | “More education time is allocated for teaching about medications” (I, N134) | “There were more teaching about medications throughout the program” (I, P214) |
|  | Structure | Alignment between pharmacology and clinical courses including the progress of the subject | “To rely on us having received sufficient and adequate education during semester 2 [the pharmacology course, at the time integrated in the physiology course], because that course is terribly important and the focus is not primarily on pharmacology. Plus [the course] is purely theoretical which isn't super-optimal for sustained learning in real life!” (I, P8) | “Have specific practical tasks within pharmacology that prepare you” (I, P237) |
|  |  | Repetition of medicinal drugs | “It's been so long since we studied pharmacology, so it would be good to have refresher lectures within each [clinical] course” (I, N114) | “More repetition during the education. Easy to forget medications even from just 6 months ago.” (I, P224) |
|  |  | Scheduled time for pharmacotherapy | “Medication took up more space in the course! By that I mean that there should be more lectures about medications, interactions, how they should be prescribed, etc” (I, P125) | “Set aside time in the schedule for studying medications!” (I, P236) |
|  |  | Mandatory medication-related learning activities during clinical courses | “it [drug therapy] was a mandatory learning activity during clinical placement” (I, P5) | “The practice quizzes were compulsory” (I, N250) |
|  | Summative assessment | Quiz assessments | “You are tested/quizzed regarding how to write prescriptions after an overview on how this is done in Melior [the electronic health record]” (I, N6) | “the summative quiz was also a good resource. This was a good way to test yourself as well” (H, P237) |
|  |  | End-of-course assessment | “In the course assessments so few scores are related to medications that one can readily achieve good results without sufficient knowledge about pharmacology...” (I, N106) | “[There were] more emphasis on pharmacology in the course assessment. Then one would have focused more on it.” (I, N251) |
| Clinical placement | Preparation | Demonstration or training of prescribing in electronic systems | “Go through how to write e-prescriptions and to work practically in the medication module” (I, P17) | ”… I could write prescriptions, prescribe medications, inject medications etc under supervision during clinical placements” (I, N206) |
|  |  | Training with electronic decision support for prescribing | “We got to learn more about where to find the information once you're a doctor because I doubt that I'll remember everything when I'm done” (I, N13) | “Thought the quizzes were helpful. I spent quite a lot of time on these and got good at using FASS [Pharmaceutical Specialities in Sweden], reklista [a regional list of recommended medications per therapeutic area], Janusmed [a national knowledge resource including, for instance, drug interactions, medications during pregnancy, medications in breast feeding]” (H, P202) |
|  |  | Auscultation during nurses’ drug dispensing and administration | ‒ | “During clinical placement at internal medicine, we hung out with a nurse for half a day. Very useful to see the handling of medications in practice. As a doctor, you don't see medication very often, except as a line in Melior [the electronic medical record system]. More like that on more courses! (hang out with a nurse)” (I, P202) |
|  | Participation | Listen to the physicians’ reasoning about medication | “Clinical placements. Much discussion about medications” (H, P1) | “I learn most from hearing how doctors reason about medication choices” (H, P203) |
|  |  | Supervisors who encourage engagement in medication-related work tasks | “Doctors were more "interested" in my thoughts during the rounds. To have a more clearly defined supervisor during clinical placements - that is, that the supervisor's position as supervisor should be clearer” (I, P13) | “My supervisors at the clinical placement who asked a lot of questions and involved us students in the medication-related work tasks” (H, P228) |
|  |  | Discuss pharmacotherapy with physicians | “Mainly during clinical placements where helpful registrars described various patients' medication lists and why these medications were chosen” (H, P9) | “Discussed [patients’] current pharmacotherapy with supervisors at clinical placements & why one medication was chosen & not another. Also, went through how to clinically weigh one medication against another when they are actually very similar" (H, P217) |
|  | Performance | Conduct medication reviews | “To work independently with patients' pharmacotherapy and carry out medication reviews (under supervision)” (H, N2) | “During practice [clinical placement] when I completed a medication review” (H, P225) |
|  |  | Write medication discharge summaries | ‒ | “Write an inpatient discharge summary and a patient friendly medication summary” (I, N242) |
|  |  | Training practically on the entire prescription process | ‒ | “It would have been helpful to do everything myself from the start. Like: here is a patient, these are the symptoms. Which medications? Where do you click in Melior [the electronic medical record system]? Talk to the patient. All steps of the way” (I, P219) |
|  |  | Student-led clinics with decisions on medications | ‒ | “I think that the student-run outpatient clinics I did during internal medicine was very helpful for training decisions on which treatment the patient should receive and suggesting doses. In addition, you had the opportunity to practice how to technically use the medication module [in the electronic medical record system], which we don't do at all at the regular clinical placements” (I, P209) |
| Theoretical teaching | Content | Clarify the importance of medications in the professional role as a physician | “If one was informed about prescriptive authority” (I, N16) | “There was more clarity about pharmacotherapy being fundamental [for the medical profession]” (I, N237) |
|  |  | Basic pharmacology | “We learned more about mechanisms of action, adverse drug reactions and drug interactions” (I, P19) | “The pharmacology part of physiology [pharmacology and physiology integrated in one course]” (H, N240) |
|  |  | Substance level | “More frequent use of generic medication names during education. It is difficult now that we know medication classes, not the specific [substance] names” (I, N15) | “…and that you often know medication classes but not the names of individual medications => don't recognize the medications in the patients’ medication list” (I, N210) |
|  |  | Practically useful medication knowledge in education | “During lectures, review regarding how to practically proceed when, for example, prescribing medications” (H, P3) | “More useful information from the education so that you don't [only] hope to learn this at clinical placements” (I, P201) |
|  |  | Teaching that simplifies reality | “Helpful with lectures that address practical recommendations” (H, N2) | “More quick reference guides with algorithms or cheat sheets were handed out” (I, N250) |
|  |  | Clinical course literature that includes pharmacotherapy | ‒ | “Course literature that more clearly describes [drug] treatment” (I, N205) |
|  | Format | Connection to patients and diseases | “during clinical placements when doctors discussed it [pharmacotherapy] in specific patient cases” (H, N108) | “Seeing patients in real life and going through their medications is by far the best way to understand how “pharmacotherapy” medications work in practice” (H, P211) |
|  |  | Case-based teaching about medications | “We had more case reviews with a focus on medication and dosage” (I, P12) | “You were given patient cases where you received relevant background information & then had to look up what kind of medications you want to prescribe” (I, P205) |
|  |  | Lectures | “Maybe [if] you had a separate lecture where you just go through different medications, side effects, interactions etc so you can sort them better in your mind” (I, P25) | “That more is included about medications in lectures during the clinical courses” (I, N253) |
|  |  | Practice quizzes | “I think short quizzes on Canvas [the digital learning platform] related to each lecture/area would have been helpful - simple and easily accessible. I think these should be voluntary in order not to create stress for those who do not learn very well that way” (I, N11) | “I enjoyed the medication quizzes and wish every clinical course included these elements. It feels like you learn in a more structured way then and that you don't forget to read about medications in FASS [Pharmaceutical Specialities in Sweden]” (I, P235) |
|  |  | Digital learning platform | ‒ | “More medication education, does not have to be live lectures, could be helpful with some material on Canvas [the digital learning platform] that you can review on your own if interested” (I, N247) |
|  |  | Simulation of clinical situations | “The simulation was also helpful for learning emergency doses of e.g. Stesolid [diazepam]” (H, N28) | “More simulation opportunities, with a focus on choosing medication and dose” (I, N244) |
|  |  | Student-activating teaching | “More seminars and discussions about dosages where it's not just one person who states the dosage and then it's done” (I, P27) | “Maybe some more IRL teaching about the subject [pharmacotherapy], not a lecture but something where you yourself are more active” (I, P232) |
|  |  | Formative test about pharmacotherapy | “We were given such a [pharmacotherapeutic] test regularly every semester with typical cases you can come across during the foundation period” (I, P122) | “Fun to do a [pharmacotherapeutic] test without pressure” (H, P218) |
|  |  | Varying forms of teaching | “Helpful to have different forms of teaching interleaved - lectures on site, home studies, quizzes and clinical placements” (H, N23) | “More and more varied teaching activities relating to medications” (I, N243) |
| Student responsibility | Theoretical learning | | “I am studying more about interactions/side effects. Continue to work to become more independent in practice [during clinical placements]” (I, N2) | “The studying I did myself helped the most” (H, P219) |
|  | Practical learning | | “… You were allowed to work very independently and to be involved in both the rounds and conversations with other doctors about pharmacotherapy for different patients. … You were also allowed to enter the medications [in the medication module of the electronic medical record] the patients would use at the ward (signed by the supervisor, of course)” (I, P19) | “… I did the medication quizzes and more medication reviews at the clinical placements” (I, N235) |

*Answers to the question “Elements that were helpful…” are referred to as helpful components (H), and answers to the question “To prepare me better …” are referred to as improvement potential (I). P denotes that the respondent answered questions regarding the psychiatry course, and N denoted the neurology course; numbers represent unique participants
