# Supplementary material for: Helpful if “Medication took up more space in the course!” – a mixed methods study about pharmacotherapeutic knowledge and digital quizzes for learning and assessment in the medical programme
Source: BMC Med Educ. 2026 Apr 30;26:706. doi: 10.1186/s12909-026-09292-7 (PMC13130682; doi:10.1186/s12909-026-09292-7)
Supplement: Supplementary file 2 — Additional file 2: Questionnaire. [file 12909_2026_9292_MOESM2_ESM.docx]

**Participant number_____**

**Knowledge of pharmacotherapy**

This questionnaire consists of two parts. Part 1 contains 20 knowledge questions (either Psychiatry or Neurology). Part 2 contains 17 background questions.

**Part 1 Knowledge questions**

*Circle the correct answer. Answer all questions, even those where you are unsure of the answer. Only circle one answer per question. You should not use any aids when answering the questions.*

**Psychiatry**

**1. Your patient is a 54-year-old woman with depression. A few days ago, she started treatment with sertraline. Now she is experiencing anxiety in the evenings, and you add oxazepam to help her during the first few weeks of the antidepressant treatment.**

**You explain to your patient that she should avoid the combination of oxazepam and alcohol because:**

1. Alcohol causes delayed absorption of oxazepam, which prolongs its half-life
2. Oxazepam inhibits the breakdown of alcohol
3. Alcohol enhances the central nervous system effects of oxazepam
4. Alcohol increases the absorption of oxazepam, which shortens the half-life

**2. A patient is being treated with oxazepam, metoprolol, simvastatin, amitriptyline, warfarin, and digoxin.**

**Besides oxazepam, which of these medications do you suspect is MOST LIKELY to contribute to drowsiness?**

1. Amitriptylin
2. Digoxin
3. Metoprolol
4. Simvastatin

**3. A 49-year-old man is hospitalized due to a hip fracture. He has been drinking around eight beers a day for many years. One day after admission, he develops increased autonomic symptoms and becomes increasingly agitated and restless.**

**Which of the following medications is MOST appropriate to administer to reduce these symptoms?**

1. Haloperidol
2. Oxazepam
3. Thiamin (vitamin B1)
4. Disulfiram

**4. A 55-year-old woman has taken 30 capsules of oxycodone (10 mg) in a suicide attempt. She arrives at the emergency department 3 hours later. She has a respiratory rate of 11 breaths per minute, a pulse of 46, a blood pressure of 80/50 mm Hg, and an oxygen saturation of 92%. She is drowsy and difficult to arouse.**

**Which is the MOST appropriate treatment for this patient?**

1. Intravenous acetylcysteine
2. Intravenous flumazenil
3. Gastric lavage with 50 grams of activated charcoal
4. Intravenous naloxone

**5. A 33-year-old woman with depression is about to start on an SSRI. Her medical history reveals a past bulimia nervosa, and she is therefore concerned about weight gain as a side effect of the SSRI.**

**Which SSRI is MOST appropriate for this patient?**

1. Citalopram
2. Escitalopram
3. Sertraline
4. Fluoxetine

**6. A 25-year-old patient with schizoaffective disorder presents with high fever for several days and generally deteriorated condition.**

Vital signs:

Blood pressure 130/70 mmHg, pulse 85/min, respiratory rate 12/min, oxygen saturation 99%, temperature 39.5°C

Current medications:

Clozapine 150 mg, 1x2

Lithium 42 mg, 2x2

Zopiclone 7,5 mg at night

**Which of the following blood tests is MOST urgent considering the pharmacological treatment?**

1. CRP
2. Liver function tests
3. Lithium concentration
4. White blood cell count

**7. A 44-year-old man being treated for depression arrives at the emergency department in the afternoon because he is feeling unwell. He is on vacation and forgot his medication at home, so he could not take his morning dose. He has been taking the same medication for several years and has been stable in his mental health. He cannot remember which medication he is taking.**

**Which of the following is the most likely medication?**

1. Fluoxetine
2. Methylphenidate
3. Mirtazapine
4. Venlafaxine

**8. A 35-year-old previously healthy man has recently been diagnosed with ADHD and as part of the treatment, he will be started on central stimulants. Which parameter/parameters need to be examined before starting this treatment and then monitored at each adjustment of the medication?**

1. AST and ALT
2. ECG
3. Weight, pulse, and blood pressure
4. Na, K, and Ca

**9. A 30-year-old man receiving medication-assisted treatment for opioid dependence (MATOD) is prescribed buprenorphine/naloxone.**

**What happens if he incorrectly takes his medication by injecting it and why does this not occur when he takes the medication sublingually?**

1. Naloxone blocks the euphoric effect of buprenorphine when taken intravenously. Sublingually, naloxone is inactive due to first-pass metabolism.
2. Buprenorphine is active sublingually but inactive when taken intravenously because it is a partial antagonist.
3. Naloxone is only absorbed intravenously and then potentiates the effect of buprenorphine, which can lead to respiratory depression.
4. Naloxone is active sublingually but inactive when taken intravenously because it is a partial antagonist.

**10. You meet a 40-year-old woman who is seeking help for neuropathic pain and depression.**

**Which of the following medications is MOST reasonable if you want to prescribe a medication that is indicated for both neuropathic pain and depression?**

1. Amitriptylin
2. Escitalopram
3. Vortioxetine
4. Mirtazapine

**11. A 39-year-old woman has unexpectedly become pregnant. Based on the date of her last menstruation, she has calculated that she is at week 5+4. She has had recurrent depressions and has been stable for a few years. Currently, she is being treated with the following medications: sertraline (100 mg once daily), zopiclone (7.5 mg at night), and mirtazapine (15 mg at night). She is eager to have a child but fears a relapse into depression.**

**Which of the following options is MOST REASONABLE at this time regarding her pharmacotherapy?**

1. To inform the woman that there is a risk that this treatment has harmed the fetus and therefore recommend an ultrasound in a few weeks, followed by a decision on whether to terminate the pregnancy.
2. To discontinue all medication and reintroduce it if the depression returns.
3. To replace zopiclone with another sleeping pill.
4. To taper off mirtazapine and zopiclone.

**12. A 45-year-old patient arrives at the emergency department due to chest pain after a paddle training session during his lunch break. You see that he is being treated with lithium and the concentration is 1.4 mmol/L. Should the evening dose be adjusted?**

1. No, the concentration should be measured 12 hours after intake.
2. Yes, a high concentration of lithium can cause chest pain.
3. No, the level is within the normal reference range and should not be adjusted.
4. Yes, during an investigation for a possible heart attack, all medications should be discontinued.

**13. You have a patient who is prescribed fluoxetine for depression. You now want to prescribe hydrochlorothiazide for hypertension. Which of the following electrolyte disorders is MOST likely to occur with this combination of medications?**

1. Hypokalemia
2. Hyperkalemia
3. Hyponatremia
4. Hypernatremia

**14. A 54-year-old man presents to the emergency department. He reports loss of appetite and nausea. Additionally, he feels dizzy and is worried about his shaky hands. The patient is taking his medications as prescribed and there have been no recent changes. He started using ibuprofen six days ago because he sprained his ankle.**

Physical examination:

Vital signs: Blood pressure 141/80 mmHg, pulse 81/min, respiratory rate 14/min, oxygen saturation 98%, temperature 37 degrees Celsius.

Extremities: Fine tremor

Weight: 112 kg

Otherwise, no abnormalities in the examination

Medical history:
Bipolar disorder type 1 for 20 years. Essential hypertension and hypercholesterolemia for five years.

Current medications:

Amlodipine 5mg, once daily

Atorvastatin 20mg, once daily

Lithium 400 mg, twice daily

Zolpidem 10 mg, one tablet as needed

Ibuprofen 400 mg, three times daily

Preliminary lab results:

Serum creatinine: 160 micromol/L (ref 62-105), eGFR: 45mL/min/1.73m2 (ref >60)

**Which of the following drug interactions is MOST likely to have caused the symptoms?**

1. Amlodipine och lithium
2. Atorvastatin och lithium
3. Ibuprofen och lithium
4. Zolpidem och lithium

**15. A 44-year-old woman arrives at the emergency department. She is anxious, dizzy, sweaty, tremulous, and appears slightly confused. An accompanying relative reports that the patient has taken her medications as prescribed and has not taken any other over-the-counter medications or herbal remedies. The patient was prescribed tramadol yesterday after injuring her shoulder.**

Physical examination:

Blood pressure 100/50 mmHg, pulse 120/min, respiratory rate 27/min, oxygen saturation 96%, temperature 38.5 degrees Celsius
Neurological examination reveals dilated pupils, nystagmus, muscle rigidity, and clonus

Medical history:
Obsessive-compulsive disorder, treated with tablets for 20 years. Essential hypertension, treated with tablets for one month.

Current medications:

Amlodipine 5mg, once daily

Sertraline 100mg, twice daily

Zopiclone 7.5 mg, on tablet as needed

Tramadol 50 mg, 1-2 tablets 3-4 times daily

**Which of the following drug interactions is MOST likely to have caused the symptoms?**

1. Amlodipine och sertraline
2. Amlodipine och tramadol
3. Sertraline och tramadol
4. Zopiclone och tramadol

**16. You are in a medical emergency department where you meet a 22-year-old man who arrives with his parents because he suddenly had difficulty speaking due to severe muscle cramps in his jaw and tongue. He recently started contact with a psychiatric outpatient clinic where he began a new medication three days ago. What is the most likely diagnosis and medication category?**

1. ADHD and central stimulants
2. Bipolar disorder and lithium
3. Depression and SSRIs
4. Schizophrenia and antipsychotics

**17. You are working at a GP practice and meet a 30-year-old man who has been suffering from sleep disorders for several years. He has tried various pharmacological treatment options with varying effects.**

**He is now seeking urgent care because he is very worried after he, in his sleep, wallpapered his living room last night. He has no memory of the incident and is shaken. The patient denies taking any medication other than the recently prescribed sleep medication. He had 1-2 glasses of wine with dinner but denies any other substance intake.**

Physical examination:

The examination shows slightly elevated blood pressure and pulse but is otherwise unremarkable. You suspect a medication side effect, sleepwalking

**Which of the following sleep medications is MOST likely to cause the described symptoms?**

1. Zolpidem
2. Melatonin
3. Clomethiazole
4. Propiomazine

**18. A 67-year-old man comes to the GP practice for a follow-up. He has hypertension, hyperlipidemia, and recurrent depressions. Five years ago, he had a myocardial infarction. Since his depression has not improved despite long-term treatment, quetiapine was added one month ago. No new symptoms.**

Vital signs:

Blood pressure 125/80 mmHg, pulse 70/min, respiratory rate 15/min, oxygen saturation 98%.

Current medications:

Acetylsalicylic acid 75 mg, once daily

Hydrochlorothiazide 50 mg, once daily

Simvastatin 40 mg, once daily

Citalopram 40 mg, one daily

Quetiapine 200 mg, once daily in the evening

**Which of the following side effects should you particularly consider during today’s consultation?**

1. Prolonged QTc interval
2. Liver impairment
3. Increased blood pressure
4. Agranulocytosis

**19. A 37-year-old woman with schizophrenia comes to the outpatient clinic and mentions that she has not had her period for a couple of years. She is concerned because she and her husband have been discussing having children. She is not using any contraceptives.**

Current medications:

Risperidone 4 mg, once daily

Sertraline 100 mg, once daily

Zopiclone 7,5 mg, one tablet as needed

**Which lab test needs to be ordered?**

1. Prolactin
2. Serum concentration of risperidone
3. Hemoglobin
4. PEth

**20. You are working in a psychiatric ward where a 42-year-old woman is being treated for anorexia nervosa. When you admitted her two days ago, the following was noted in her status**

BMI: 14 kg/m^2^, Blood pressure 90/55 mmHg, pulse 50/min, temperature 35.5 degrees Celsius

**A nurse on the ward reports today that the night duty doctor prescribed a medication for the patient’s increasing anxiety and motor restlessness. Now her pulse rate has dropped to 41/min.**

**Which medication is MOST likely responsible for the decrease in pulse rate?**

1. Propiomazine
2. Mirtazapine
3. Buspirone
4. Olanzapine

**Neurology**

**1. A patient is being treated with oxazepam, metoprolol, simvastatin, amitriptyline, warfarin, and digoxin. Besides oxazepam, which of these medications do you suspect is MOST LIKELY to contribute to drowsiness?**

1. Amitriptyline
2. Digoxin
3. Metoprolol
4. Simvastatin

**2. A 44-year-old woman has unexpectedly become pregnant. Based on the date of her last menstrual period, she has calculated that she is at week 5+4. She has hypertension, asthma, pollen allergy, and epilepsy with bilateral tonic-clonic seizures, seizure-free for three years. She uses the following medications: candesartan, inhalation fluticasone, lamotrigine, and during the pollen season, desloratadine tablets and mometasone nasal spray.**

**Which of the following options is MOST REASONABLE at this time regarding her pharmacotherapy:**

1. To inform the woman that there is a risk that this treatment has harmed the fetus and therefore recommend an ultrasound in a few weeks and then consider whether to terminate the pregnancy.
2. To inform the woman that it is important for her to continue with these medications because the fetus’s development benefits from the mother being well-treated.
3. To discontinue lamotrigine, to be reintroduced if she experiences epileptic seizures again, and continue with the other medications unchanged.
4. To replace candesartan with another antihypertensive medication but continue with the same dose of lamotrigine and monitor lamotrigine concentrations.

**3. A 79-year-old man with chronic atrial fibrillation has been treated with apixaban since the fibrillation was discovered after he suffered an ischemic stroke in the left hemisphere. Two months after his stroke, he comes to the emergency room due to a witnessed epileptic seizure with focal onset and generalization. It is assessed as post-stroke epilepsy, and he is treated with carbamazepine. He is now admitted to the hospital after waking up with weakness on the left side. A CT scan shows a new infarction in parts of the right MCA territory, and CT angiography shows a corresponding distal occlusion. The patient improves during the hospital stay and is discharged after five days. Which statement below is MOST REASONABLE?**

1. Another oral anticoagulant than apixaban is warranted due to a new stroke during ongoing treatment.
2. If careful monitoring of the patient’s INR and APTT had been done, the infarction could have been avoided.
3. The patient should be prescribed another antiepileptic medication to avoid future strokes due to drug interaction.
4. Cognitive effects of antiepileptic treatment have affected the patient’s adherence to apixaban treatment.

**4. A patient is using oxcarbazepine for epilepsy and amlodipine for high blood pressure. You want to add hydrochlorothiazide due to insufficient blood pressure control. Which of the following electrolyte disorders is MOST LIKELY to occur with this medication combination?**

1. Hypokalemia
2. Hyperkalemia
3. Hyponatremia
4. Hypernatremia

**5. A 32-year-old woman visits the GP practice for prescription renewal and follow-up of thyroid tests. She mentions that she and her husband are planning on having a baby. She wonders if it is safe to use the current medications during pregnancy.**

| Present illnesses | Since 2005: Myoclonic epilepsy with 3-spike-and-wave pattern on EEG. Seizure-free since 2008 when she started her current epilepsy treatment.  Since 2011: Dysmenorrhea  Since 2015: Hypothyroidism  Since 2018: Recurrent depression |
| --- | --- |
| Medications | Sertraline 100 mg daily  Levothyroxine 100 micrograms daily  Folic acid 5 mg daily  Paracetamol 500 mg, 1-2 tablets as needed, up to three times daily  Valproate 500 mg twice daily |
| Lab values | TSH unchanged at 0.6 mU/L one week ago. (Ref 0.3-4.0) |

**Which information is MOST REASONABLE to give to the patient regarding her medications and potential pregnancy?**

1. Valproic acid should be switched to another antiepileptic medication because it interacts with levothyroxine, risking high thyroxine levels during pregnancy.
2. It is important for the fetus to have good seizure control, and the medication should not be changed as long as she takes folic acid to protect against birth defects.
3. Valproic acid should be switched to another antiepileptic medication because the medication poses a risk of birth defects.
4. The medication can continue unchanged, but if the patient becomes pregnant, an increase in the dose of valproic acid is required to provide adequate seizure control.

**6. A 71-year-old woman is admitted to the hospital due to community-acquired pneumonia. During the morning rounds, the nurse reports that the patient is confused and disoriented, and that this has been occurring with varying intensity over the past day.**

| Physical examination | Disoriented to time and place, appears confused.  Vital signs: Blood pressure 120/75 mmHg, pulse 75/min regular, respiratory rate 16/min, oxygen saturation 96% with 1L oxygen via nasal cannula, temperature 36.6 °C  No other new findings at examination except for rattling on auscultation of the left lung as expected from the pneumonia. |
| --- | --- |
| Present illnesses | Essential hypertension for 10 years  Chronic atrial fibrillation for 6 years  Parkinson’s disease for 4 years |
| Medications | Dabigatran 150 mg twice daily  Benzylpenicillin 1 g three times daily  Enalapril 10 mg daily  Levodopa/carbidopa 100/25 mg, 1 tablet at 8 AM, 12 PM, 16 PM, and 20 M  Metoprolol extended release 100 mg daily |
| Lab values | CRP 66.7 mg/L (ref < 10 mg/L)  Otherwise, normal lab values |

**Which statement is MOST REASONABLE regarding the above patient?**

1. Haloperidol should not be prescribed together with benzylpenicillin
2. Haloperidol should not be prescribed together with metoprolol
3. Haloperidol should not be prescribed to patients with Parkinson’s disease
4. Haloperidol should not be prescribed to patients older than 65 years

**7. A 70-year-old man comes to the emergency room due to increasing confusion. His daughter explains that this started yesterday. Two days ago, he began treatment with a new medication prescribed by his general practitioner “for blood pressure, or maybe because of his urinary urgency?”.’ The daughter is unsure which.**

| Physical examination | Vital parameters: Blood pressure 155/85 mmHg, pulse 92/min, respiratory rate 18/min, temperature 37.0 °C  Abdomen: Auscultated with normal bowel sounds. Palpable suprapubic resistance.  Otherwise, normal physical examination. |
| --- | --- |
| Present illnesses | Tablet-treated hypertension for 15 years  Tablet-treated hypercholesterolemia for 10 years  Urinary urgency disturbing sleep for 6 months  Started investigation of cognitive impairment 3 months ago |
| Medications | Metoprolol extended-release tablet 100 mg daily  Simvastatin 40 mg daily  Some new unknown medication |
| Lab values | P-creatinine 110 micromole/L (ref 60-105 micromole/L)  eGFR 64 mL/min/1.73m2 (>60)  No other abnormalities in lab results |
| Other examination | Urine bladder, ultrasound: 510 ml |

**NPÖ [the Swedish tool for nationally integrated medical records] is currently down, but which of the following possible medications can BEST EXPLAIN the newly arisen problems?**

1. Amlodipine
2. Lisinopril
3. Tolterodine
4. Spironolactone

**8. Which of the following options is MOST REASONABLE when it comes to measuring concentrations of antiepileptic medications in the blood?**

1. Samples should always be taken 2 hours after the last dose.
2. Concentration measurement is particularly valuable if the therapeutic range is wide.
3. It is not useful to measure concentrations if the medication has non-linear kinetics.
4. Measurement can provide information about compliance.

**9. A 62-year-old man presents at the GP practice with tremor in both hands. The problem started a year ago, and the tremors are most prominent when he eats or writes. He reports no issues with slowness, impaired balance, or other involuntary movements.**

| Physical examination | Neurological: You find bilateral tremors in the upper extremities when the patient holds his arms straight out. No rigidity or bradykinesia during diadochokinesia, and nothing else is revealed during the finger-nose test.  Physical examination: Otherwise, normal. |
| --- | --- |
| Previous and present illnesses | High blood pressure for 15 years  Elevated blood lipids treated for 13 years  Head trauma with subdural hematoma, conservatively treated 4 years ago  Post-traumatic epilepsy for 4 years  Depression for 2 years |
| Medications | Fluoxetine 20 mg daily  Hydrochlorothiazide 12.5 mg daily  Losartan 50 mg daily  Simvastatin 20 mg daily  Valproic acid 500 mg twice daily |
| Lab values | Normal findings |

**Specify which two medications are most likely contributing to his tremors.**

1. Fluoxetine
2. Hydrochlorothiazide
3. Losartan
4. Simvastatin
5. Valproic acid

**10. A man in his 80s visits the GP practice after fainting while sitting at the dinner table the day before. He recovered quickly after getting to the floor but reports dizziness when standing up and after climbing the stairs to the second floor.**

| Physical examination | Vital parameters: Blood pressure 130/82, pulse 80 lying down, 85/60, pulse 105 after three minutes standing. Temperature 37.0 °C.  Neurological: Bilateral slowness and bradykinesia with fatigue phenomena in hands and feet. Reduced facial expression. |
| --- | --- |
| Previous and current conditions | Subendocardial myocardial infarction 8 years ago  Elevated blood lipids discovered at that time  Parkinson’s disease diagnosed 3 years ago  Prostatism for 5 years |
| Medications | Acetylsalicylic acid 75 mg daily  Simvastatin 40 mg daily  Finasteride 10 mg daily  Alfuzosin 10 mg daily  Levodopa/benserazide 100/25 mg 0.5 tablet 3 times daily |
| ECG | Normal findings |
| Lab values | Normal findings |

**Which of the patient’s medications is MOST LIKELY to contribute to the observed orthostatic hypotension?**

1. Acetylsalicylic acid
2. Simvastatin
3. Finasteride
4. Alfuzosin
5. Levodopa/benserazide

**11. You meet a 32-year-old woman at the GP practice. She is planning a long trip to Asia and wants to discuss vaccination. She has pollen allergy and has been treated for multiple sclerosis with annual infusions of a monoclonal antibody, rituximab, for the past 3 years. Which statement is MOST REASONABLE?**

1. Due to immunosuppressive treatment, all vaccination is contraindicated.
2. The woman should be vaccinated with attenuated vaccines.
3. Vaccination risks reducing the effectiveness of rituximab.
4. Vaccination within the next 6 months after rituximab infusion is unlikely to provide good infection protection.

**12. A 71-year-old previously essentially healthy woman comes for a follow-up visit 2 months after suffering a TIA with transient paresis of the right arm and leg. During hospitalization at the time of the incident, both hypertension and type 2 diabetes were diagnosed, and she was prescribed several medications. For the past month, she has had leg pain, which has prevented her from walking as much as she usually does, and in the past week, she has also developed pain in her upper arms. She wonders if it could be a side effect of one of her medications.**

**Which of the following medications would be FIRST suspected as the cause of her symptoms?**

1. Clopidogrel
2. Atorvastatin
3. Enalapril
4. Metformin

**13. An 83-year-old man is on his second day of care in the stroke unit after being treated with IV thrombolysis and thrombectomy for an embolism proximal to the left middle cerebral artery. Upon arrival, he had aphasia and right-sided hemiparesis. These symptoms have largely regressed, and a follow-up CT scan of the brain the next day shows neither bleeding nor demarcation of infarction. He has been previously treated with metoprolol and enalapril for mild heart failure. Telemetry monitoring has shown episodes of normofrequent atrial fibrillation, and you are now considering dabigatran to reduce the risk of new embolizations from the heart.**

**Which of the following tests do you absolutely have to have results for before you can start treatment with dabigatran?**

1. NT-proBNP
2. Na, K, and Creatinine
3. Creatinine and eGFR
4. ECG to check QT interval

**14. A 79-year-old woman with anticoagulant-treated atrial fibrillation presents as a stroke alert around 11 PM after a witnessed sudden onset of left arm paresis and neglect at 9:45 PM. An emergency CT scan of the brain shows no bleeding or fresh infarction. Blood pressure is 165/88.**

**Which of the following statements is MOST REASONABLE?**

1. Thrombolysis can be considered after reversal with a monoclonal antibody if the patient is on anticoagulant treatment with dabigatran.
2. Thrombolysis can be considered after reversal with a prothrombin complex concentrate if the patient is on anticoagulant treatment with apixaban.
3. Thrombolysis can be considered if INR < 1.7 and the patient is on anticoagulant treatment with dabigatran.
4. Thrombolysis can be considered after reversal with prothrombin complex concentrate if the patient is on anticoagulant treatment with warfarin.

**15. A 57-year-old woman with diabetes, hypertension, and gastroesophageal reflux issues suffers a TIA and is prescribed secondary prophylactic treatment with clopidogrel. Which of her regular medications should be considered for substitution due to pharmacokinetic reasons in light of the clopidogrel treatment?**

1. Metformin
2. Omeprazole
3. Candesartan
4. Atorvastatin

**16. "A 32-year-old woman, adopted from Vietnam, contacts the clinic with general malaise and a rash on her trunk, upper arms, and face after starting a new treatment for epilepsy. Which medication do you suspect she received without prior HLA testing?**

1. Levetiracetam

2. Lacosamide

3. Valproic acid

4. Carbamazepine

**17. A nursing home resident with epilepsy and a long-standing seizure-free period develops slowed movements, impaired speech, and worsening of cognitive function. A CT scan of the brain shows atrophy compared to an examination five years ago. Which antiepileptic medication do you ensure the patient is NOT taking?**

1. Topiramate
2. Valproic acid
3. Lamotrigine
4. Levetiracetam

**18. A 24-year-old woman with epilepsy is treated with, among other medications, lamotrigine and presents at the accident and emergency department after a seizure. The concentration of lamotrigine in her blood is significantly lower than her usual values. Which option is LEAST LIKELY as an explanation for the woman’s decreased lamotrigine levels?**

1. Pregnancy
2. Poor compliance
3. Adjunctive treatment with carbamazepine
4. Adjunctive treatment with valproic acid

**19. As the night duty doctor at the medical clinic, you have just checked in with the night nurse on the stroke ward. As you step out into the stairwell, a nursing assistant calls you back to room 3, where a 68-year-old man, admitted four days ago for a stroke with left-sided hemiparesis, is staying. You return and find him displaying clonic jerks in his extremities, blood-tinged froth in his mouth, and rightward gaze deviation with frequent jerking in that direction. The nursing assistant reports that he had jerking in his left hand for half an hour prior to the onset of the generalized seizure, which has now been going on for five minutes. The patient has a peripheral venous catheter with a Ringer’s acetate drip infusing.**

**According to the medical record, the patient has a 20-year history of hypertension and benign prostatic hyperplasia. His weight was 82 kg yesterday.**

| Medications | Clopidogrel 75 mg once daily  Enalapril 20 mg once daily  Atorvastatin 10 mg daily  Finasteride 5 mg once daily |
| --- | --- |

**You determine that he is having an ongoing epileptic seizure. Given that it seems to have lasted more than 5 minutes without subsiding, you decide to administer seizure-breaking treatment. Which treatment is MOST REASONABLE?**

1. Diazepam 5 mg/ml, rectal administration of 2 ml
2. Diazepam injection solution 5 mg/ml, intravenous administration of 10 ml
3. Midazolam injection solution 5 mg/ml, intramuscular injection of 1 ml
4. Diazepam injection solution 5 mg/ml, intravenous injection of 2 ml

**20. A 72-year-old woman has been diagnosed with trigeminal neuralgia at the GP practice. She started treatment with gabapentin capsules 300 mg three times a day. Despite this, her symptoms have escalated over the past week, and she is seeking emergency care this Saturday morning due to frequent pain attacks. You examine her in the emergency room and find no neurological abnormalities except for slightly altered sensory quality on the right side over the maxilla. During the examination, she experiences an intense pain attack, which also occurs when she drinks water. Despite the pain, she has managed to feed herself orally, and you do not identify an immediate need for hospital care. Which treatment adjustment is MOST REASONABLE?**

1. Add extended-release oxycodone 5 mg twice daily
2. Add amitriptyline 25-50 mg at night
3. Increase gabapentin dose to a maximum of 1200 mg three times daily
4. Add ibuprofen 400 mg three times daily

**Part 2 Background questions**

Including questions that reflect pharmacotherapy within the framework of the psychiatry/neurology [the alternative at issue presented in the questionnaire] course.

| *Indicate (by checking one box per question) to what extent you agree with the following statements (on a scale from “Totally disagree” to “Totally agree”)* | *Totally disagree* | |  | |  | *Totally agree* | |
| --- | --- | --- | --- | --- | --- | --- | --- |
| **1.** I feel confident in my knowledge of common psychiatric/neurological medications regarding mechanisms of action, effects, and side effects. | □ | □ | | □ | □ | | □ |
| **2.** I feel confident in my knowledge of common psychiatric/neurological medications regarding dosage. | □ | □ | | □ | □ | | □ |
| **3.** I feel confident in my knowledge of the principles of pharmacokinetics related to common psychiatric/neurological medications. | □ | □ | | □ | □ | | □ |
| **4.** I feel confident in performing medication reviews. | □ | □ | | □ | □ | | □ |
| **5.** I feel confident in writing medication discharge summaries. | □ | □ | | □ | □ | | □ |
| **6**. I am familiar with the procedures/systems of writing prescriptions for medications. | □ | □ | | □ | □ | | □ |
| **7**. I am familiar with the procedures/systems of prescribing medications to inpatients | □ | □ | | □ | □ | | □ |
| **8**. I have sufficient knowledge to evaluate clinical trials, for example to avoid being misled by marketing. | □ | □ | | □ | □ | | □ |
| **9**. I have sufficient knowledge of overall regulations related to medications, such as the Pharmaceutical Reimbursement System. | □ | □ | | □ | □ | | □ |
| **10.** I think that the medical programme has so far prepared me well for my role as a doctor when it comes to treating patients with medications. | □ | □ | | □ | □ | | □ |

**11**. I am □ a woman

□ a man

□ non-binary

□ other alternative

□ uncertain

□ unwilling to respond

**12**. I am □ ≤24 years

□ >24 years

□ unwilling to respond

**13**. I **□** have a PhD degree

**□** do not have a PhD degree

□ am unwilling to respond

**14**. I have studied the course in basic pharmacology:

□ at the University of Gothenburg

□ at another university

□ unwilling to respond

**15**. This semester, I study:

□ both the neurology and the psychiatry courses at the University of Gothenburg

□ only one of the courses above

**16**. The following components of the psychiatry/neurology course have helped me grow into the role of a physician when it comes to the responsibility for patients’ medication management. (Answer in free text)

__________________________________________________________________________________

__________________________________________________________________________________

__________________________________________________________________________________

__________________________________________________________________________________

__________________________________________________________________________________

__________________________________________________________________________________

__________________________________________________________________________________

**17**. To prepare me better for the responsibility that comes with the prescribing rights as a licensed physician, it would be helpful if… (Answer in free text)

__________________________________________________________________________________

__________________________________________________________________________________

__________________________________________________________________________________

__________________________________________________________________________________

__________________________________________________________________________________

__________________________________________________________________________________

__________________________________________________________________________________
